# Spexin Enhances Bowel Movement through Activating L-type Voltage-dependent Calcium Channel via Galanin Receptor 2 in Mice

**DOI:** 10.1038/srep12095

**Published:** 2015-07-10

**Authors:** Cheng-yuan Lin, Man Zhang, Tao Huang, Li-ling Yang, Hai-bo Fu, Ling Zhao, Linda LD Zhong, Huai-xue Mu, Xiao-ke Shi, Christina FP Leung, Bao-min Fan, Miao Jiang, Ai-ping Lu, Li-xin Zhu, Zhao-xiang Bian

**Affiliations:** 1 Lab of Brain and Gut Research, School of Chinese Medicine, Hong Kong Baptist University, Hong Kong SAR, China; 2 YMU-HKBU Joint Laboratory of Traditional Natural Medicine, Yunnan Minzu University, Kunming, China; 3 School of Pharmaceutical Sciences, Guangzhou Medical University, Guangzhou, China; 4 Institute of Basic Research in Clinical Medicine, China Academy of Chinese Medical Sciences, Beijing, China; 5 University at Buffalo School of Medicine and Biomedical Sciences, 3435 Main Street, Buffalo, NY,USA

## Abstract

A novel neuropeptide spexin was found to be broadly expressed in various endocrine and nervous tissues while little is known about its functions. This study investigated the role of spexin in bowel movement and the underlying mechanisms. In functional constipation (FC) patients, serum spexin levels were significantly decreased. Consistently, in starved mice, the mRNA of spexin was significantly decreased in intestine and colon. Spexin injection increased the velocity of carbon powder propulsion in small intestine and decreased the glass beads expulsion time in distal colon in mice. Further, spexin dose-dependently stimulated the intestinal/colonic smooth muscle contraction. Galanin receptor 2 (GALR2) antagonist M871, but not Galanin receptor 3 (GALR3) antagonist SNAP37899, effectively suppressed the stimulatory effects of spexin on intestinal/colonic smooth muscle contraction, which could be eliminated by extracellular [Ca^2+^] removal and L-type voltage-dependentCa^2+^ channel (VDCC) inhibitor nifedipine. Besides, spexin dramatically increased the [Ca^2+^]i in isolated colonic smooth muscle cells. These data indicate that spexin can act on GALR2 receptor to regulate bowel motility by activating L-type VDCC. Our findings provide evidence for important physiological roles of spexin in GI functions. Selective action on spexin pathway might have therapeutic effects on GI diseases with motility disorders.

Spexin is a recently identified neuropeptide composed of 14 amino acids, which is highly conserved in different vertebrates^1^,^2^,^3^. Tissue distribution studies in rat and goldfish showed that spexin is widely expressed in skin, respiratory system, digestive system, urinary system, reproductive system, nervous system and endocrine system^3^,^4^, indicating that spexin may play multiple functions. It has been reported that spexin can modulate cardiovascular and renal function and nociception in mice^5^. Recent studies in goldfish suggested that spexin can suppress the serum luteinizing hormone (LH) level^6^ and appetite^3^. Furthermore, it is also involved in weight regulation by reducing adipocyte uptake of long chain fatty acids in rats and mice^7^.

Many neuroendocrine hormones are crucial factors for gastrointestinal (GI) functions including bowel movement^8^,^9^,^10^. As a new member of this neuroendocrine peptide family, spexin is speculated to play important roles in GI function for the following reasons: firstly, spexin is widely expressed in different gut areas in rats^4^; secondly, the GALR2/3 which are supposed to be activated by spexin are also located in gastric, intestinal and colonic tissues in rats^11^ and other vertebrates^12^; thirdly, spexin can induce the contraction of gastric tissue *in vitro*^2^, which is the direct evidence current available. Therefore, further studies are necessary to investigate the roles of spexin in GI tract.

Recently, it was reported that spexin can activate human GALR2 and GALR3 receptors with high binding affinities *in vitro* by ligand-receptor interaction assay^13^. GALR2/3 receptors are implicated in diverse biological functions, in which the central nervous system functions controlling memory, seizure, pain, anxiety and mood disorder are the most intensely investigated^14^,^15^,^16^,^17^. In rats, GALR2 receptor can mediate galanin-induced jejunal contraction^18^, indicating that spexin may also play roles in bowel movement via galanin receptors.

The present study investigated the effects of spexin on bowel movement and further examined possible mechanisms for spexin effects on bowel movement. We provide evidence that spexin can stimulate both intestinal and colonic movement through L-type Voltage-dependent Calcium Channel activation via GALR2.

## Results

### Serum spexin levels in FC patients

**Figure 1:**
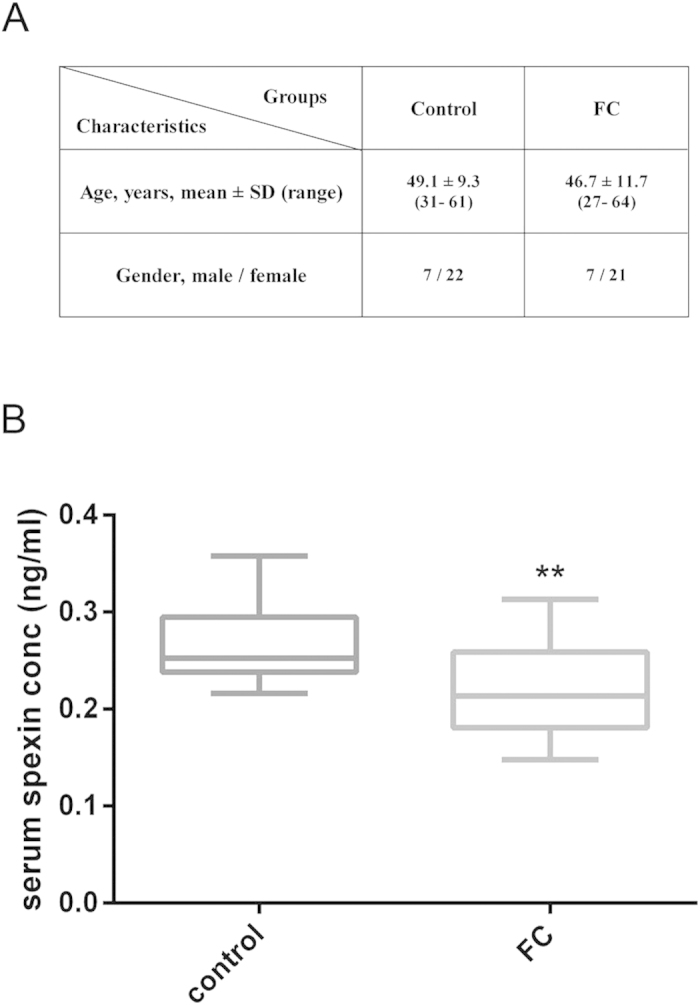
Serum spexin levels in FC patients. (**A**) Characteristics of FC patients and control groups. The diagnosis of FC was based on the Rome III criteria. (**B**) Changes of serum spexin levels in FC patients. Control group n = 31, FC group n = 29. Data are expressed as means ± SEM, Statistical differences between individual groups were evaluated using Student’s *t* test. **P < 0.01 compared to control group.

The baseline characteristics of patients and healthy control groups were described in Fig. 1A, and there was no significant difference between two groups (*P* = 0.2). The mean age of healthy group was 49.58 ± 1.56 with 7 male and 24 female and the mean age of FC group was 46.29 ± 2.03 with 7 male and 22 female. Significant decrease of serum spexin level was observed in FC patients (0.225 ± 0.009 ng/ml, n = 29, *P* = 0.0024) compared with healthy control (0.271 ± 0.011 ng/ml, n = 31) (Fig. 1B).

### Spexin mRNA expressions in the intestine and colon of starvation mice

**Figure 2:**
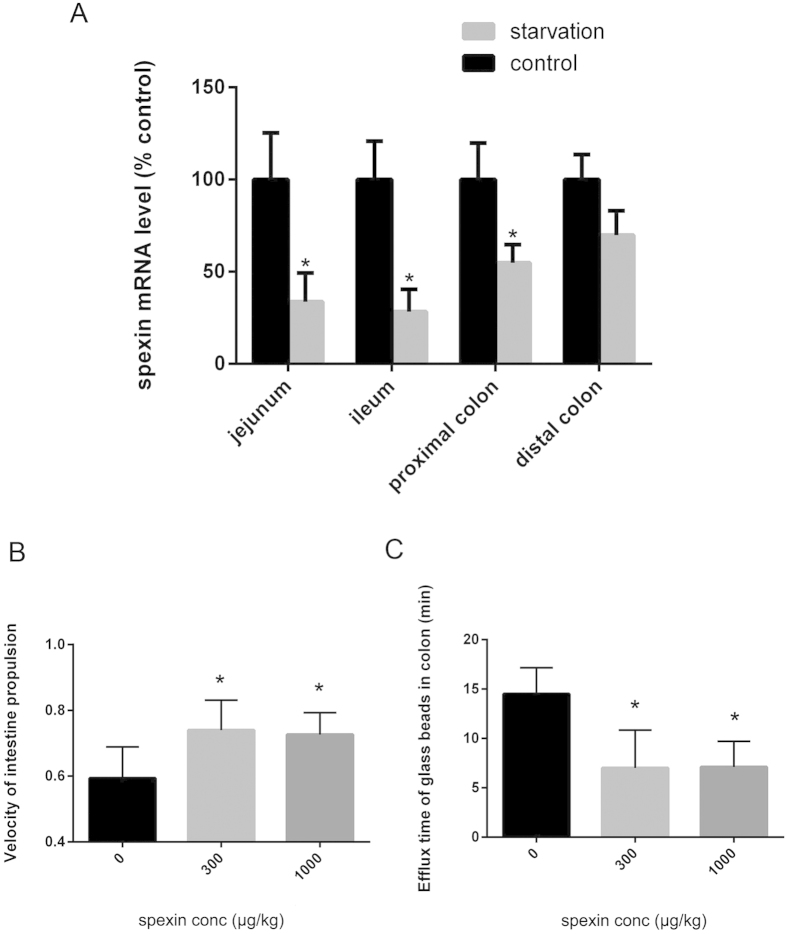
Effects of spexin on the motor activity of gastrointestinal tract in mice *in vivo*. (**A**) Expression level of spexin mRNA in intestine and colon of mice under the starvation condition. Mice were starved for 24 hours and then the total RNA of proximal colon, distal colon, jejunum and ileum were prepared for real-time PCR of spexin mRNA. (**B**) Effect of intraperitoneal (*ip*) spexin on propulsion of the carbon powder in the intestine. Saline or spexin (300 μg/kg and 1000 μg/kg) was injected *ip* and mice were placed in individual cages without water and food for 20 minutes. Then 0.2 ml 10% powdered carbon suspended in 5% gum arabic was intragastric administered. 15 minutes later, the mice were sacrificed and the distance of carbon-ink from the pylorus to the most distal point of the charcoal was recorded. (**C**) Effect of spexin (*ip* injection) on the efflux time of glass beads in the colon. A single 3-mm colored plastic bead was inserted into the distal colon (2.5 cm past the anus) with a lubricated plastic rod and then saline or spexin (300 μg/kg and 1000 μg/kg, respectively) were administrated by *ip* injection. The expulsion time of the bead for each mice was monitored. Statistical differences between individual groups were evaluated using One way ANOVA. *P < 0.05 and **P < 0.01 compared with paired saline-treated controls.

As shown in Fig. 2A, the mRNA level of spexin in jejunum and ileum after starvation stress were significantly lower at 33.7 ± 15.6% (n = 8, P = 0.012) and 28.3 ± 12.1% (n = 8, P = 0.006) that of the control group. Meanwhile, spexin mRNA expression in proximal colon and distal colon decreased to 54.8 ± 9.9% (n = 8, P = 0.045) and 69.8 ± 13.3% (n = 8, P = 0.103) that of the paired sham-operated controls. In contrast, the mRNA level of galanin significantly increased in ileum, proximal colon and distal colon, while galanin receptor (GALR1, GALR2 and GALR3) levels were elevated in colon but not intestine of starved mice (see Supplementary Fig. S5 online).

### Effects of spexin on bowel movement ***in vivo***

Spexin (300 μg/kg and 1 mg/kg) injection increased the propulsion speed of intestine content in mice. Compared with the vehicle (0.61 ± 0.09), *ip* injection of 300 μg/kg spexin and 1 mg/kg spexin significantly increased the intestinal propulsion velocity to 0.73 ± 0.11 (n = 12, P = 0.007) and 0.72 ± 0.06 (n = 12, P = 0.005), respectively (Fig. 2B). In the study of distal colonic transit measurement, spexin (300 μg/kg and 1 mg/kg, ip) significantly decreased the colonic transit time to 7.15 ± 1.66 min (n = 10, P = 0.038) and 7.17 ± 1.24 min (n = 10, P =0.043), respectively, compared with 14.79 ± 3.67 min in vehicle-treated mice (Fig. 2C). These results indicate a positive correlation between spexin level and bowel movement.

### Effects of spexin on bowel movement ***in vitro***

**Figure 3:**
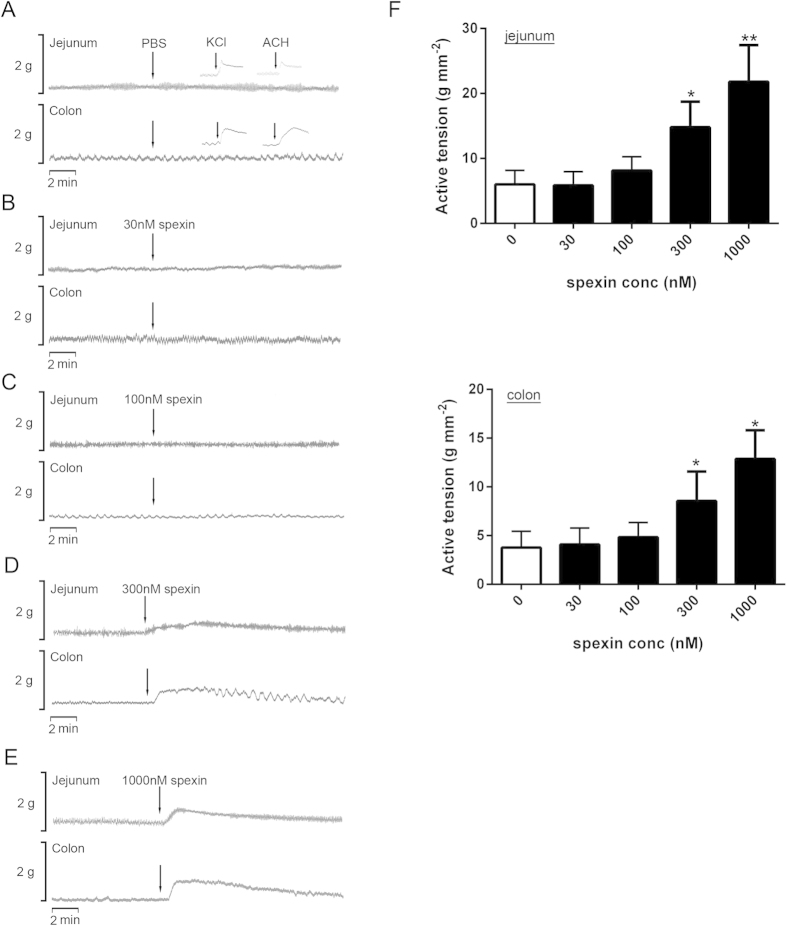
Effects of spexin on the contraction of mice jejunum and colon in the organ bath system *in vitro*. The tissues were allowed to equilibrate for 1 hour and the mechanical activities of smooth muscles in the presence of PBS (**A**) or spexin 30 nM (**B**) 100 nM (**C**) 300 nM (**D**) and 100 nM (**E**) were recorded using the POWERLAB system and CHART5 software. KCl and ACH treatment were used as positive controls. (**F**) The active tensions in the colon and jejunum were calculated. Statistical differences between individual groups were evaluated using One way ANOVA. **P < 0.01 and ***P < 0.001 compared with paired PBS-treated controls.

To further confirm the effects of spexin on bowel movement, *in vitro* organ-bath studies were performed. In the results, spexin (30 nM–1 μM) could induce the contractile response of both jejunum and colon in a dose-dependent manner. In jejunum, 30 nM and 100 nM spexin did not alter the active tension (5.9 ± 0.7 g mm-2 and 8.1 ± 0.8 g mm-2, respectively, n = 6) (Fig. 3B,C) compared with PBS-treated control group (6.0 ± 0.8 g mm-2) (Fig. 3A). However, 300 nM and 1 μM spexin could significantly increase the active tension to 14.8 ± 1.4 g mm-2 (n = 6, P = 0.002) and 21.8 ± 2.0 g mm-2 (n = 6, P < 0.001), respectively (Fig. 3D,E). Similarly, 30 nM and 100 nM spexin did not significantly affect the colonic smooth muscle contraction (4.1 ± 0.8 g mm-2 and 4.8 ± 0.7 g mm-2, respectively, n = 6) (Fig. 3B,C) compared with PBS-treated control group (3.8 ± 0.8 g mm-2) (Fig. 3A), while significant stimulation effects were found upon 300 nM and 1 μM spexin treatment with active tension of 8.6 ± 1.4 g mm-2 (n = 6, P = 0.003) and 12.9 ± 1.3 g mm-2 (n = 6, P < 0.001), respectively (Fig. 3D,E). KCl and acetylcholine (ACH) treatment were used as positive controls. The statistical cartograms of the spexin effects on jejunum and colon contraction were shown in Fig. 3F.

### Modeling of Mouse Galanin Receptor-Spexin Complexes

To demonstrate that spexin may also interact with GALR2/3 in mouse and to gain insights into the important interactions involved, a combined approach including homology modeling, molecular dynamics (MD) and molecular docking was used (see Supplementary Fig. S1 online). We generated three-dimensional (3D) models for spexin (see Supplementary Fig. S2 online) and mouse GALR2/3 (see Supplementary Fig. S4 online) via homology modeling and MD. We also built the mouse GALR-Spexin complex models with flexible molecular docking (see Supplementary Fig. S5 online).These results revealed that spexin fitted the binding site of mouse GALR2/3 well, and several hydrogen bonding and hydrophobic contacts between spexin and GALR2/3 were predicted. While they need to be confirmed by binding assay and mutation experimentation, these results suggest that spexin may also activate GALR2/3 in mouse.

### Effects of GALR2/3 receptor antagonism on spexin-induced bowel tissue contraction ***in vitro***

**Figure 4:**
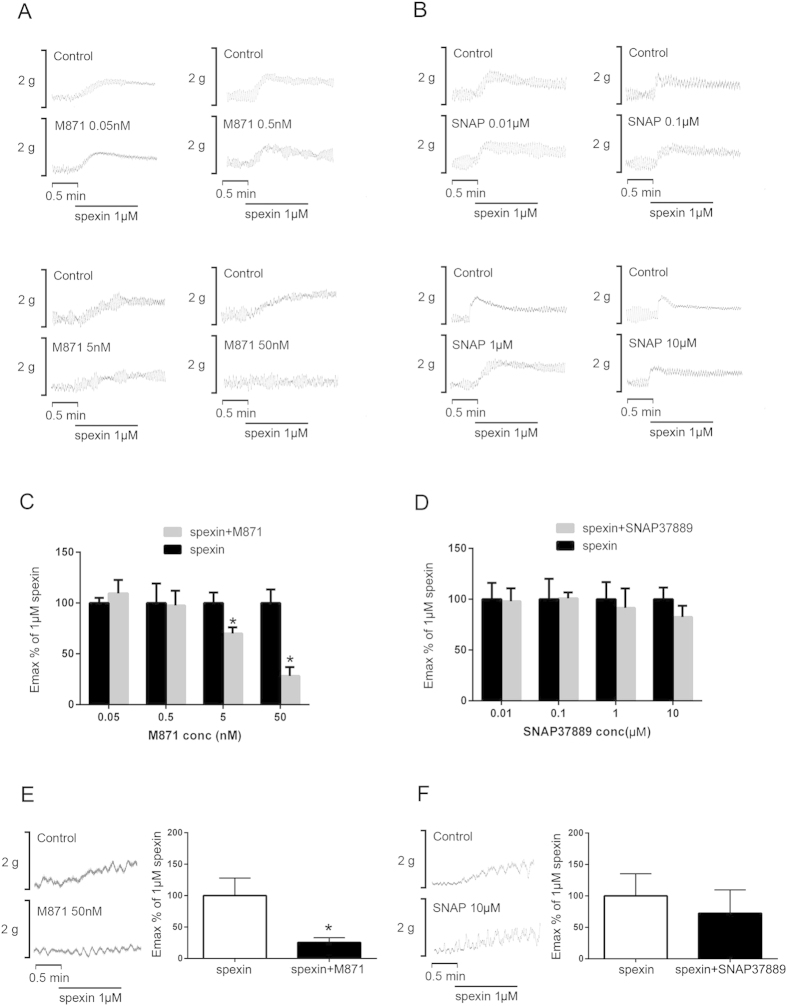
Effects of GALR2/3 antagonists on spexin-induced intestinal and colonic motility. The jejunum tissues were allowed to equilibrate for 1 hour and then treated with GALR2 antagonist M871 (0.05–50 nM, A &C) and GALR3 antagonist SNAP37889 (0.01–10 μM, B&D). The colon tissues were allowed to equilibrate for 1 hour and then treated with GALR2 antagonist M871 (50 nM, E) and GALR3 antagonist SNAP37889 (10 μM, F). 30 minutes later, the tissues were treated with spexin (1 μM) and the mechanical activities were recorded using the POWERLAB system and CHART5 software. The Emax% of 1 μM spexin in jejunum were calculated. Statistical differences between individual groups were evaluated using One way ANOVA. *P < 0.05 and **P < 0.01 compared with paired saline-treated controls.

To test if GALR2/3 are involved in the stimulatory effects of spexin on intestinal and colonic smooth muscle contraction, the jejunum segments were subjected to GALR2 antagonist M871 and GALR3 antagonist SNAP37889 for 30 min with increasing doses (0.05 nM–50 nM), respectively. In this case, the spexin-induced jejunum smooth muscle contraction was suppressed by M871 in a dose-dependent manner (Fig. 4A,C) but not affected by SNAP37889 (Fig. 4B,D). The minimum effective dose for the blockage was noted at 5 nM (E_max_% is 69.9 ± 6.2%, n = 6, P = 0.045),while the E_max_% of 50 nM M871 is 28.2 ± 8.7%, n = 6, P = 0.003). Similarly, GALR2 antagonist M871 could suppress the contractile amplitudes of colon segments to 25.6 ± 7.5% (n = 6, P = 0.0046) of the maximum effect induced by spexin in normal conditions (Fig. 4E). GALR3 antagonist SNAP37889, however, did not exhibit significant effect on spexin-induced smooth muscle contraction in mice colon (Fig. 4F).

### Role of Ca^2+^-dependent pathways in spexin-induced intestinal and colon contraction ***in vitro***

**Figure 5:**
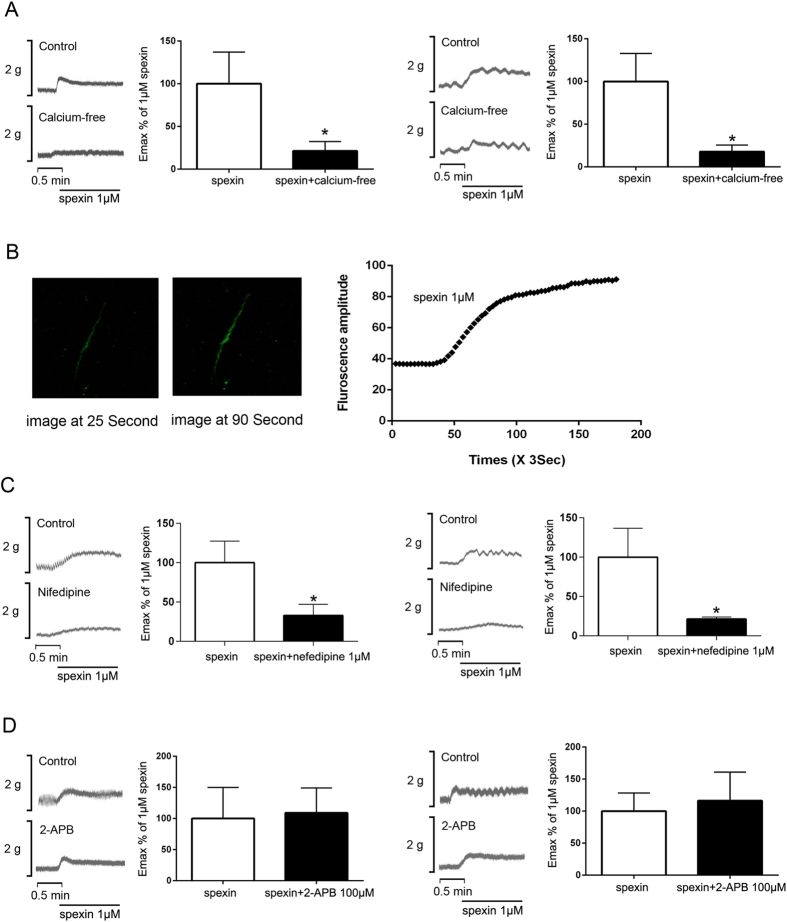
Effects of Ca^2+^ influx and release on spexin-induced intestinal and colonic motility. (**A**) The jejunum and colon tissues were allowed to equilibrate for 1 hour and then the nutrition buffers were replaced with Ca^2+^ free buffer supplemented with 1mM EGTA. 30 minutes later, the tissues were treated with spexin (1 μM) and the mechanical activities were recorded using the POWERLAB system and CHART5 software. The Emax% of 1 μM spexin in jejunum and colon were calculated. Statistical differences between individual groups were evaluated using Student’s *t* test. **P < 0.01compared with paired saline-treated controls. (**B**) Primary colonic smooth muscle cells were isolated, preloaded with the Ca^2+^-sensitive dye Fura-4 and challenged with 1 μM spexin. The fluorescence amplitude of Ca^2+^ signal was recorded. Further, The jejunum and colon tissues were allowed to equilibrate for 1 hour and then treated with L type-VSCC inhibitor nifedipine (1 μM, **C**) and IP3 receptor inhibitor 2-APB (100 μM, D). Thirty minutes later, the tissues were treated with spexin (1 μM) and the mechanical activities were recorded using the POWERLAB system and CHART5 software. The Emax% of 1 μM spexin in jejunum and colon were calculated. Statistical differences between individual groups were evaluated using Student’s *t* test. **P < 0.01compared with paired saline-treated controls.

In a Ca^2+^-free condition, the contractile amplitudes of jejunum and colon segments were suppressed to 21.4 ± 4.5% (n = 6, P = 0.0014) and 17.8 ± 4.5% (n = 6, P = 0.002) of the maximum effect induced by spexin in normal conditions (Fig. 5A). In parallel studies, [Ca^2+^]i was monitored in isolated mice colonic smooth muscle cells after spexin treatment. As shown in Fig. 5B, spexin was effective in triggering a rapid rise in intracellular free [Ca^2+^] level in smooth muscle cells. Besides, blocking L-type VSCC by the dihydropyridine inhibitor nifedipine (10 μM) could effectively suppress spexin-induced smooth muscle contraction. After nifedipine treatments, the E_max_% of 1 μM spexin was 32.9 ± 7.1% (n = 6, P = 0.005) and 21.5 ± 1.2% (n = 6, P = 0.005) (Fig. 5C) in jejunum and colon, respectively. In contrast, IP3 receptor inhibitor 2-APB (100 μM) did not alter the spexin-induced contractile response of intestinal and colonic smooth muscle (Fig. 5D).

### Effect of spexin on intestinal and colonic contractile response with tetrodotoxin pretreatment

**Figure 6:**
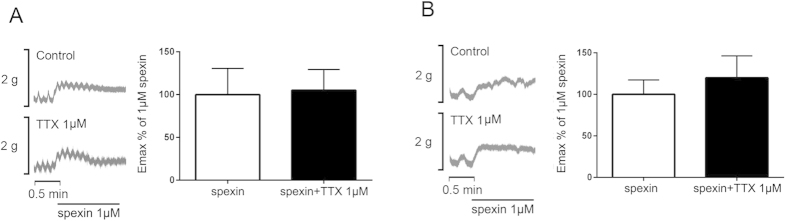
Effects of TTX on spexin-induced intestinal and colonic motility. The jejunum (**A**) and colon (**B**) tissues were allowed to equilibrate for 1 hour and TTX was then added into the nutrition buffer. 30 minutes later, the tissues were treated with spexin (1 μM) and the mechanical activities were recorded using the POWERLAB system and CHART5 software. The Emax% of 1 μM spexin in the colon and jejunum were calculated accordingly. Statistical differences between individual groups were evaluated using Student’s *t* test.

To test whether the spexin-induced bowel movement is mediated by enteric neurons, the intestinal and colonic segments were exposed to TTX (1 μM) in Krebs solution of the organ bath tubes for 30 min to block the neuronal factors in the enteric nervous system on smooth muscle contraction. Subsequently, 1 μM spexin was added into the solution of both TTX-pretreated group and control group. The amplitude of both jejunum (Fig. 6A) and colon (Fig. 6B) contraction induced by spexin showed no difference between the TTX-pretreated and the control group.

## Discussion

Although spexin is speculated to play roles in the GI disorders^2^, there was no direct evidence to support this claim. In the present study, we found that serum spexin levels were significantly decreased in FC patients (*P* < 0.01). Moreover, spexin mRNA level in the intestine and colon of starved mice showed a dramatic decrease compared with the control group. Similar to constipation, starved animals exhibit slower gut transit^19^,^20^. These results suggest that spexin is a possible regulator for gut transit especially bowel movement. To further test this hypothesis, effects of spexin on bowel movement in C57BL/6J mice model were examined. Extraneous spexin can significantly increase the propulsion of both intestine and colon in mice. Further, spexin also dose-dependently stimulated mouse intestinal and colonic smooth muscle contraction *in vitro*. So it can be concluded that spexin is a positive regulator for bowel movement, and it may play roles in the bowel motility disorder related diseases such as constipation and diarrhea.

Recently, Kim *et al*. found that spexin can activate GALR2 and GALR3 but not GALR1 through ligand-receptor interaction study *in vitro*^13^. To elucidate the interactions between spexin and GALRs from structural view, the mouse GALR2/3-Spexin complex models were built in the present study. Those modeling and simulation results implied that mSPX may be inserted into GALR2/3 via the rigid N-terminal part, which was consistent with the sequence alignment results^13^. The complex models also revealed important residues that involved in the GALR2/3-Spexin interactions, which provides hints for site-directed mutation studies.

Using GALR2 and GALR3 antagonists, we, for the first time, demonstrated that the biological functions of spexin in bowel movement regulation were mediated by GALR2 receptor but not GALR3 receptor. Although both GALR2 and GALR3 are G-protein coupled receptors, activation of the two receptors may occur with different downstream signaling events. Base on the current knowledge, G_o_/G_i_ proteins are mainly involved in the neurotransmitter-mediated calcium channel inhibition^21^. In contrast, G_q_-coupled receptors can activate the PKC and Gβγ to stimulate L-type calcium channel^22^. Ca^2+^ influx via the L-type Ca^2+^ channels or Ca^2+^ store release through IP_3_ receptors are the primary mechanisms for excitation-contraction coupling in gut smooth muscles^23^. Activation of GALR2 may evoke either inhibitory effects through *G*i/o proteins or stimulatory effects through *G*q/11 proteins^24^,^25^, while GALR3 activation mainly coupled with *G*i/o proteins^26^. So there is a possibility that spexin mainly activate GALR2 and exert stimulatory effect on smooth muscle contraction through *G*q/11 proteins in the bowel of mouse. Moreover, the expression level of GALR2 is much higher than that of GALR3 in rat intestine and colon^27^. These facts are in harmony with our findings that GALR2 instead of GALR3 mediates the spexin-induced bowel movement.

In starved mice, the mRNA levels of GALR2 and GALR3 were significantly increased in the colon tissues, which was not consistent with the changes of spexin expression. These results may be due to the increase of galanin levels in the intestine and colon of starved mice. Based on current knowledge, the effect of galanin on gastrointestinal motility are controversial with both stimulatory and inhibitory effects reported in different species, tissues and experimental conditions^28^. However, it has been reported that central and peripheral galanin could stimulate food intake^28^ and decrease leptin synthesis and secretion in rats^29^, which were in coherent with the increased plasma galanin concentration in fasted rats^30^. Thus, the expression levels of galanin receptors are most likely to be positively correlated with the increased galanin level, but not the decreased spexin level in starved mice.

It has been shown that activation of GALR2 stimulates large conductance Ca^2+^-dependent K^+^ channels through the IP3 pathway in human embryonic kidney (HEK293) cells^31^. GALR2 also plays a role in the galanin-induced contraction in the rat myometrium by stimulating both intracellular Ca^2+^ release and extracellular Ca^2+^ influx^32^. In the present study, we found that spexin-induced bowel movement could be blocked by [Ca^2+^]e removal and L-type VDCC blockade, but did not respond to the IP3 receptor antagonism, indicating that spexin may regulate bowel smooth muscle contraction mainly through Ca^2+^ influx.

Besides Ca^2+^-dependent cascades, submucosal enteric motor neurons is another important regulator for the intestinal smooth muscle contraction^33^ which can be blocked by TTX^34^. To test whether the spexin-induced bowel movement is mediated by enteric neurons, TTX was used to inhibit the enteric neuronal activity. Our results suggested that spexin stimulated bowel movement independent of neuronal action potentials.

In summary, the present study demonstrates that spexin can stimulate both intestinal and colonic movement in mice. GALR2 receptor activation and the subsequent Ca^2+^ influx mediated through L-type VDCC are involved in the signaling mechanisms of spexin-induced bowel movement. Our findings suggest that spexin is an important neuroendocrine factor in regulating GI motility and selective action on spexin pathway might have therapeutic effects on GI diseases with motility disorders including constipation and diarrhea.

## Methods

### Patients and serum samples

Twenty-nine healthy subjects and 28 FC patients were recruited from clinics of School of Chinese Medicine, HKBU. Informed consent was obtained from each patient, and the study protocol was approved by the Hong Kong Baptist University Ethics Committee on the Use of Human Subjects for Teaching and Research^35^. The clinical study was registered with an identifier (NCT01695850) in Clinical Trial.gov in 2012.

The inclusion criterial of FC patients were listed as follows: Patients were included if they had all of the following: 1) Met the diagnostic criteria for FC (Rome III)^36^; 2) Age of 18 to 65 years (inclusive); 3) Complete spontaneous bowel movement (CSBM) ≦2times/wk (CSBM is defined by feeling of complete passage of stool after defecation, rather than partial or incomplete evacuation, without the use of any laxative or enema within 24 hours)^37^; 4) Severity of constipation ≧3 points (on a 7-point scale)^38^; 5) Total symptom score ≧8 points (on a 7-point scale for constipation-related symptoms); 6) Normal colonic examination (barium enema or colonoscopy) within five years; 7) Normal liver and renal function in blood test within 3 months.

The inclusion criterial of healthy subjects were listed as follows: 1) no history of neurologic or psychological illness; 2) no history of cardiovascular, cerebrovascular, or endocrine disease; 3) no abnormal findings on body examinations; 4) no history of constipation; 5) no abnormal results of blood analysis including whole blood count, renal and liver function tests, plasma glucose test.

Blood samples were obtained from 29 healthy subjects and 28 FC patients at 9am in the morning by fasting for 12 hours. Serum spexin levels were determined using ELISA (cat. no. EK-023-81 CE; Phoenix Pharmaceuticals, Belmont, CA USA).

### Animals

Male C57BL/6J mice weighing about 20–24 g were purchased from the Laboratory Animal Services Center, The Chinese University of Hong Kong, Hong Kong. The animals were fed with a standard rodent diet *ad libitum* with free access to water and were housed in rooms maintained at 22 ± 1 °C with a 12 h light/dark cycle (lights on 6:00–18:00). Animals were acclimated to the facility for 1–2 wk before the experiments. All mice were used once for each experiment. The Animal Ethics Committees of Hong Kong Baptist University, approved all experimental protocols, in accordance with “Institutional Guidelines and Animal Ordinance” from Department of Health, Hong Kong Special Administrative Region.

### Spexin mRNA measurement in starvation mice by real-time PCR

Total 24 mice were divided into 2 groups randomly and equally. The control group was housed (4 mice per cage) and maintained on standard diet *ad libitum* with free access to water. The treatment group (4 mice per cage) was fasted for 24 hours with free access to water. And then the mice were euthanized with CO_2_, and the jejunum, ileum, proximal colon and distal colon were collected. The tissues were homogenized by Tissuelyser LT (cat. no. 85600; Qiagen, Hilden, Germany) in appropriate volume TRIZOL (cat. no. 15596018; Life technologies, Invitrogen, Carlsbad, CA, USA) and the total RNA was extracted. The cDNA was synthesized using the SuperScript® First-Strand synthesis system for RT-PCR (cat. no. 18080051; Invitrogen, Carlsbad, CA, USA) according to the manufacturer’s instruction. Quantitative real-time PCR for spexin was conducted on the ViiA™ 7 Real-Time PCR System (Applied Biosystems, Foster city, CA, USA) with Power SYBR GREEN Master Mix (cat. no. 4367659; Applied Biosystems, Foster city, CA, USA). The primer sequences are as follows: 5'-CTGGTGCTGTCTGCGCTG-3' and 5'-CTGGGTTTCGTCTTTCTGG-3'.

### Intestinal transit measurement ***in vivo***

Mice were fasted for 16 h prior to experiments, and divided into 3 groups randomly with 15 mice in each group. Spexin (300 ug/kg and 1000 ug/kg) or saline were injected intraperitoneally and mice were placed in individual cages without water and food for 20 minutes. Then 0.2 ml 10% powdered carbon suspended in 5% gum arabic was intragastrically administered. Fifteen minutes later, the mice were sacrificed by CO_2_ asphyxiation separately, and the intestines were harvested between the pylorus and the ileocecal junction. The distance of carbon-ink from the pylorus to the most distal point of the charcoal was recorded as a migration distance. The velocity of intestine propulsion is subjected to the equation: (migration distance/total length of the small intestine)×100%^39^.

### Colonic transit measurement ***in vivo***

Mice were fasted for 16 h prior to experiments, and divided into 3 groups randomly with 15 mice for each group. Under brief ether anesthesia, a single 3-mm colored plastic bead was inserted into the distal colon (2.5 cm past the anus) with a lubricated plastic rod and then spexin (300 ug/kg and 1000 ug/kg, respectively) or saline were injected intraperitoneally. The injected mice were placed in individual cages without water or food. The expulsion time of the bead for each mice was monitored following the methods in the literature^40^.

### Intestinal and colonic motility tests ***in vitro***

Organ bath system was used to test the smooth muscle motility according with the previous methods^41^. Briefly, adult male mice (20–24 g) were euthanized with CO_2_, the jejunum and colon were immediately harvested and flushed with Krebs solution (119 mM NaCl, 4.5 mM KCl, 1.2 mM MgCl_2_, 25 mM NaHCO_3_, 1.2 mM KH_2_PO_4_, 2.5 mM CaCl_2_ and 11.1 mM glucose). The organ bath was bubbled with a mixture of 95% O_2_ plus 5% CO_2_, and maintained at 37 °C. About 1 cm long piece of the tissue was longitudinally placed in the organ bath containing Krebs solution. The mechanical activity of longitudinal smooth muscle was recorded using the POWERLAB system and CHART5 software (AD instrument Ltd., Bella Vista, NSW, Australia). The tissues were allowed to equilibrate for 1 hour before the experiment with the washing step every 20 min with Krebs solution. And then, effects of spexin (cat. no. 023–81;Phoenix Pharmaceuticals, Belmont, CA USA) were tested. Acetylcholine chloride (ACh)(Cat. no. A2661; Sigma, St. Louis, MO USA) and KCl (Cat. no. P9333; Sigma-Aldrich, St. Louis, MO USA) were used as positive controls. To examine the possible mechanisms of spexin on bowel movement, TTX (cat. no. 1069; Tocris Bioscience, Bristol, UK), EGTA (cat. no. E3889; Sigma-Aldrich, St. Louis, MO USA), nifedipine (cat. no.N7634; Sigma-Aldrich, St. Louis, MO USA), 2-APB (cat. no. D9754; Sigma-Aldrich, St. Louis, MO USA), M871(cat. no. ab141159; Abcam, Cambridge, UK) and SNAP37889 (cat. no. 11L-312S; Key organics, Camelford, UK) were applied separately to block the corresponding pathway. Thirty minutes later, spexin was added to test the effect on bowel motility. The amplitude of contractions was measured and expressed as force/area (g/mm2) using the equation (force/area = grams tension/[gram wet wt/(1.05 × Lo)], with 1.05 as the density of smooth muscle) according to the previous reported method^42^. The optimal length (*L*o) was obtained by using several sections from different mice at initial stretch of 0.5 g to obtain a maximum response to 1 μm ACh^43^. The inhibitory effects of the blockers were expressed as inhibitory rate of E_max_% (E_max_ is the maximum effect induced by spexin).

### Smooth muscle cells isolation and laser confocal fluorescent imaging

Mice were euthanized with CO_2_, the colon tissue were harvested quickly. Smooth muscle cells were isolated as described previously^44^. Briefly, smooth muscle layers separated from mice colon were washed in Ca^2+^-free HBSS solution (142 mM NaCl, 5.6 mM KCl, 0.44 mM K_2_HPO4, 1.0 mM MgCl_2_, 0.34 mM Na_2_HPO4, 5.6 mM glucose, and 10 mM HEPES; pH 7.4), and then digested in PBS containing 2 mg/ml collagenase type II, 1 U/ml papain (cat. no. P4762; Sigma-Aldrich, St. Louis, MO USA), 2 mg/ml trypsin inhibitor (cat. no.T0256; Sigma-Aldrich, St. Louis, MO USA) and 0.05% BSA (cat. no. 05470; Sigma-Aldrich, St. Louis, MO USA) for 20–40 min. The digested tissue suspension was further dispersed by repeated pipetting with blunt pipettes followed by 10 minutes centrifugation with a speed of 1000 × g. The dispersed cells in the pellet were collected and washed with pre-cooled Ca^2+^-free solution. Isolated smooth muscle cells were seeded on the cover slides coated with Corning® Cell-Tak™ Cell and Tissue Adhesive reagent (cat. no. 354240; BD Biosciences, NJ, USA ) for the following [Ca^2+^]i imaging and measurement within 8 hours according the reported procedure^45^. Cells were preloaded with Ca^2+^-sensitive dye Fluo3/AM (2WM, Molecular Probes, Eugene, OR, USA) for 40 minutes in the dark at 37 °C in HBSS solution. Then the cultured cells were washed for three times with HBSS and transferred into the chamber. Single cell [Ca^2+^]i will be measured in the Leica confocal system (Leica Microsystems Heidelberg GmbH, Germany) continuously for at least 3 min before and after spexin (1 μM) treatment. Intracellular Ca^2+^ level was expressed as the florescence signals at 510 nm triggered by 488nm excitation (referred to as florescence amplitude).

### Statistical analysis

The data are presented as means ± SEM. Statistical differences between individual groups were evaluated using Student’s *t* test or one-way ANOVA. GraphPad Prism 6.0 software (GraphPad Software Inc., San Diego, CA, USA) was used for the calculations. A P value of < 0.05 was considered statistically significant.

## Additional Information

**How to cite this article**: Lin, C.-y. *et al*. Spexin Enhances Bowel Movement through Activating L-type Voltage-dependent Calcium Channel via Galanin Receptor 2 in Mice. *Sci. Rep*. **5**, 12095; doi: 10.1038/srep12095 (2015).

## Supplementary Material

Supplementary Information
