# Supplementary material for: Spexin Enhances Bowel Movement through Activating L-type Voltage-dependent Calcium Channel via Galanin Receptor 2 in Mice
Source: Sci Rep. 2015 Jul 10;5:12095. doi: 10.1038/srep12095 (PMC4498193; doi:10.1038/srep12095)
Supplement: Supplementary Information [file srep12095-s1.pdf]

SUPPORTING INFORMATION

Spexin Enhances Bowel Movement through Activating L-type Voltage-dependent Calcium Channel via galanin receptor 2 in mice

Cheng-yuan Lin[#], Man Zhang[#], Tao Huang, Li-ling Yang, Hai-bo Fu, Ling Zhao, Linda LD Zhong, Huai-xue Mu, Xiao-ke Shi, Christina Fung-ping Leung, Bao-min Fan, Miao Jiang, Ai-ping Lu, Li-xin Zhu, Zhao-xiang Bian[*]

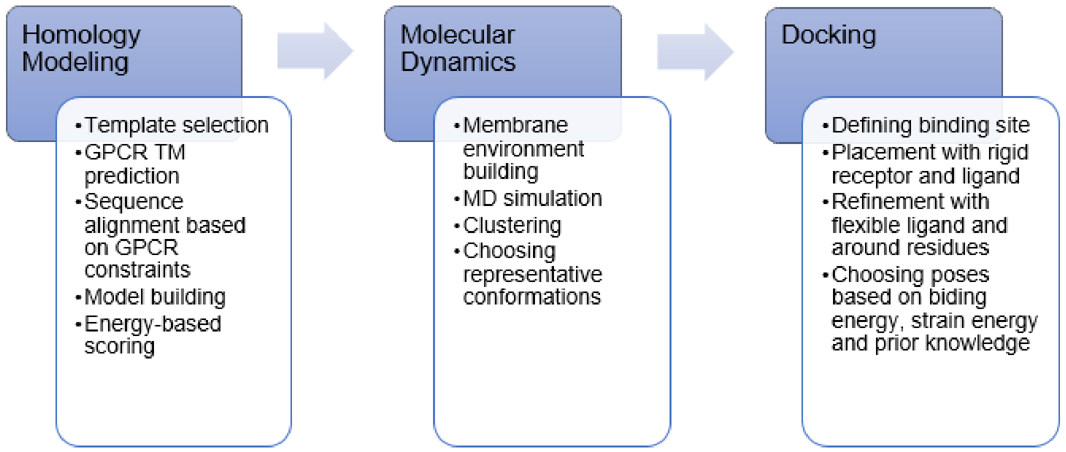

Supplementary Figure S1. The combined approach to build the mouse GALR2/3-Spexin complexes model. Homology modeling was used to build the initial structures of mSPX and mouse GALR2/3. The resulted structures were further refined with molecular dynamics. The representative conformations were used for flexible docking. The final complex models were chosen based on prior knowledge, binding energy and ligand strain energy.

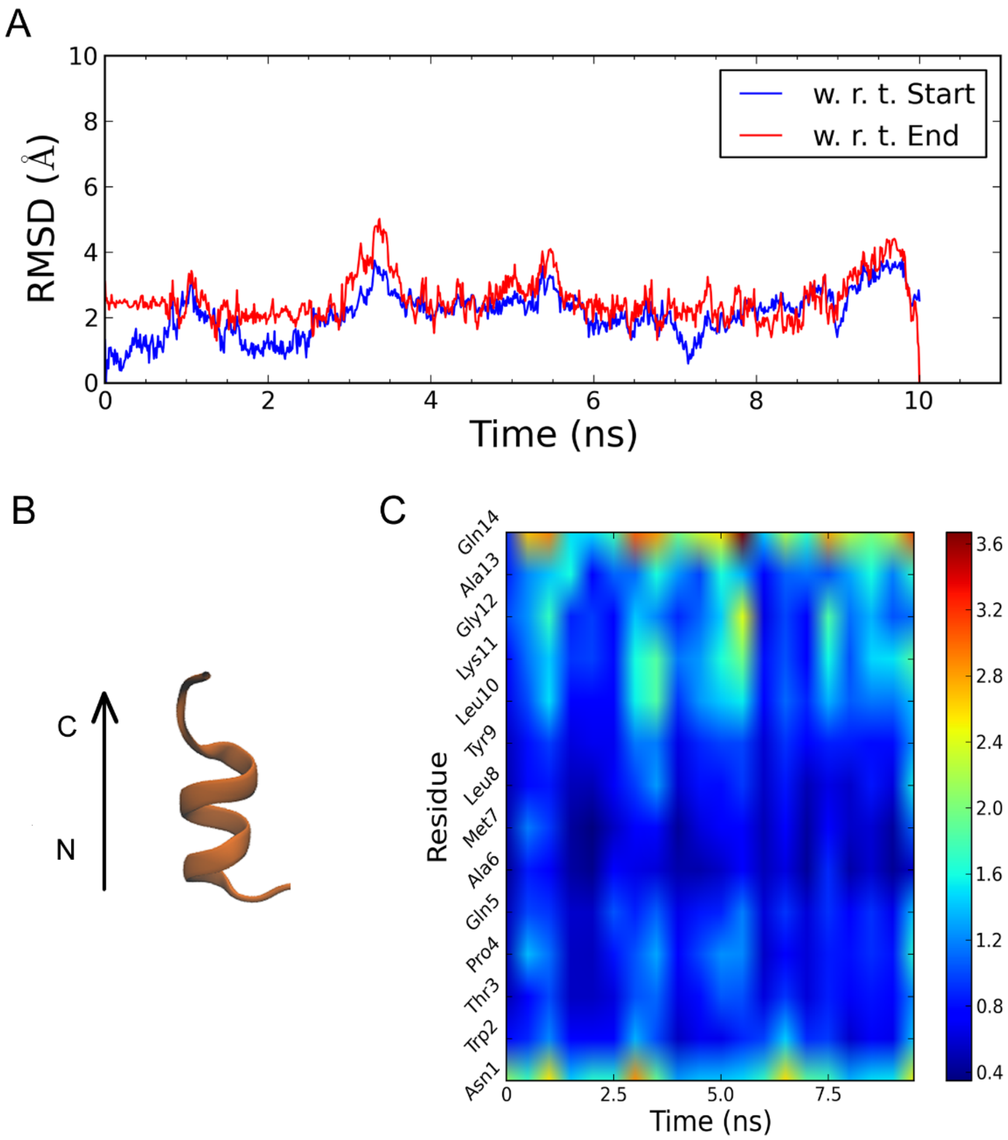

Supplementary Figure S2. The mouse spexin model. A, the 3D structure of mSPX built from homology modeling and refined by 10 ns MD simulation. B, the C_α RMSD evolution of mSPX (except Gly1 and Gln14) over 10 ns with regard to the start (blue) and end (red) structures. C, the C_α root mean structural fluctuation (RMSF) of each residue of mSPX during the simulation.

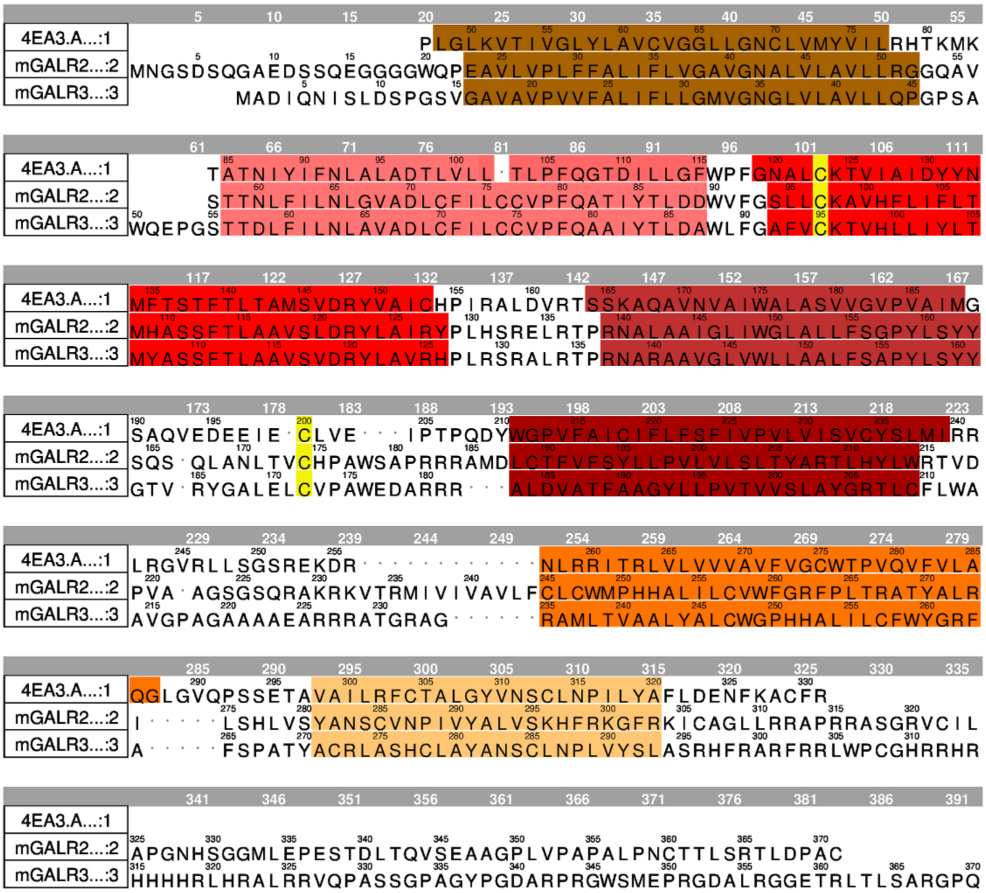

Supplementary Figure S3. The sequence alignment between mouse GALR2/3 and template 4EA3.A. The 7 trans-membrane (TM) regions were annotated and colored. The cysteines which could form disulfide bond were colored in yellow.

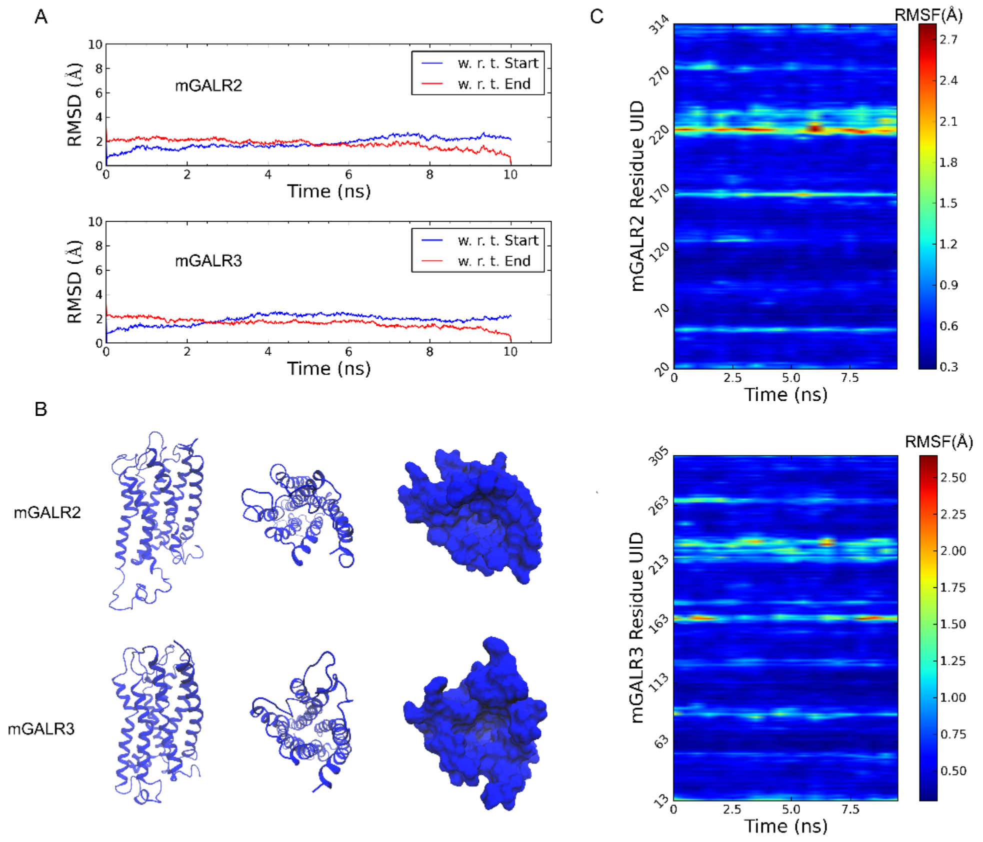

Supplementary Figure S4. The mouse GALR2/3 models. A, the RMSD evolution of mGALR2/3 in 10 ns MD simulation with regard to the start (blue) and end (red) structures. B, the overall structure of mGALR2/3. From left to right, the front view rendered in cartoon, the top view rendered in cartoon, the top view rendered in surface. C, the RMSF of each residuein mGALR2/3 over 10 ns MD simulation.

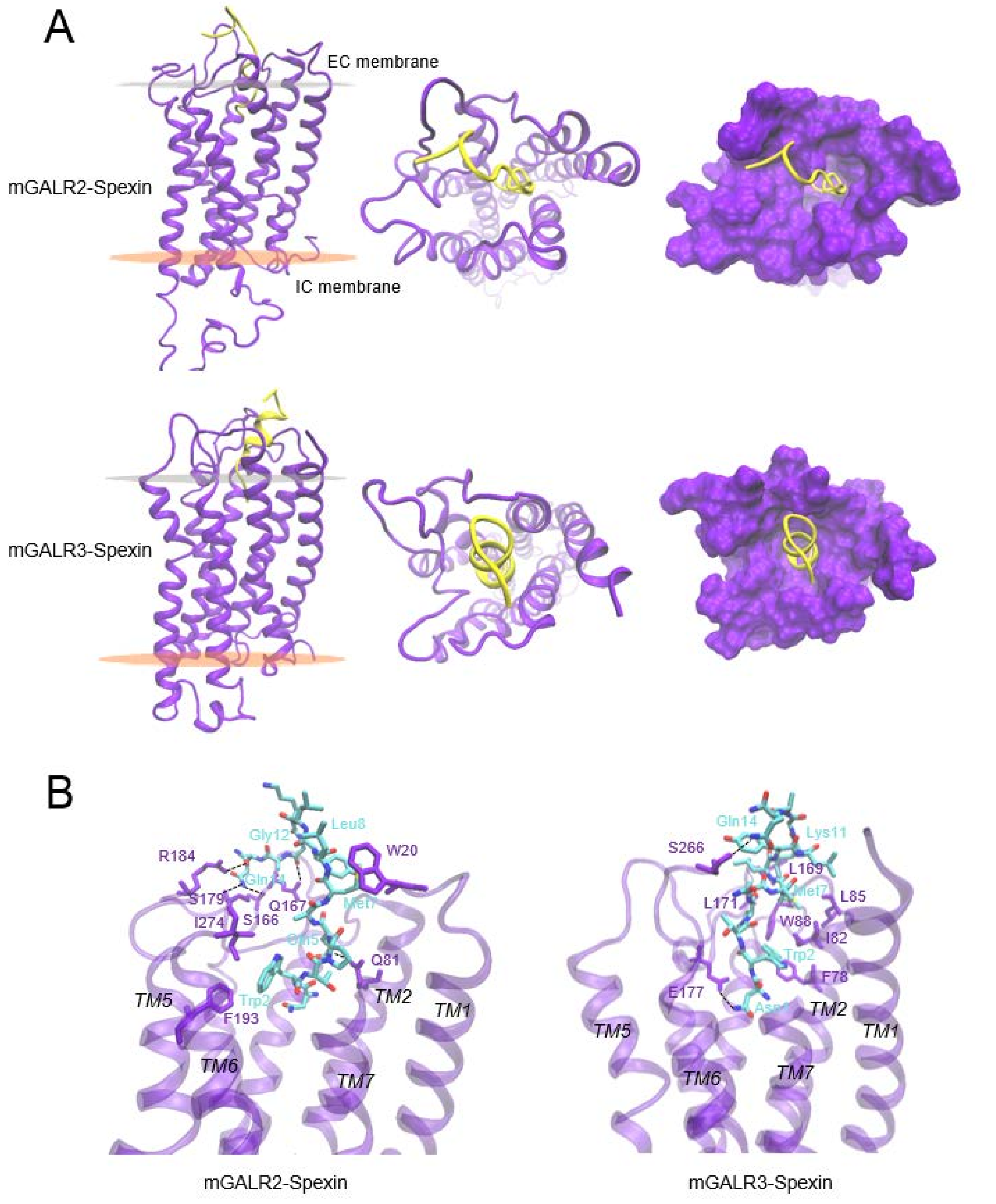

Supplementary Figure S5. The mouse GALR2/3-Spexin Complexes Models. A,the best docking poses of mGALR2/3-Spexin. From left to right, the front view rendered in cartoon, the top view rendered in cartoon, the top view rendered in surface. The mGALR2/3 was colored in purple and spexin was colored in yellow. B, the interactions between mGALR2/3 and spexin. The receptor residues involved in interaction were labeled in purple and the spexin residues were labeled in cyan.

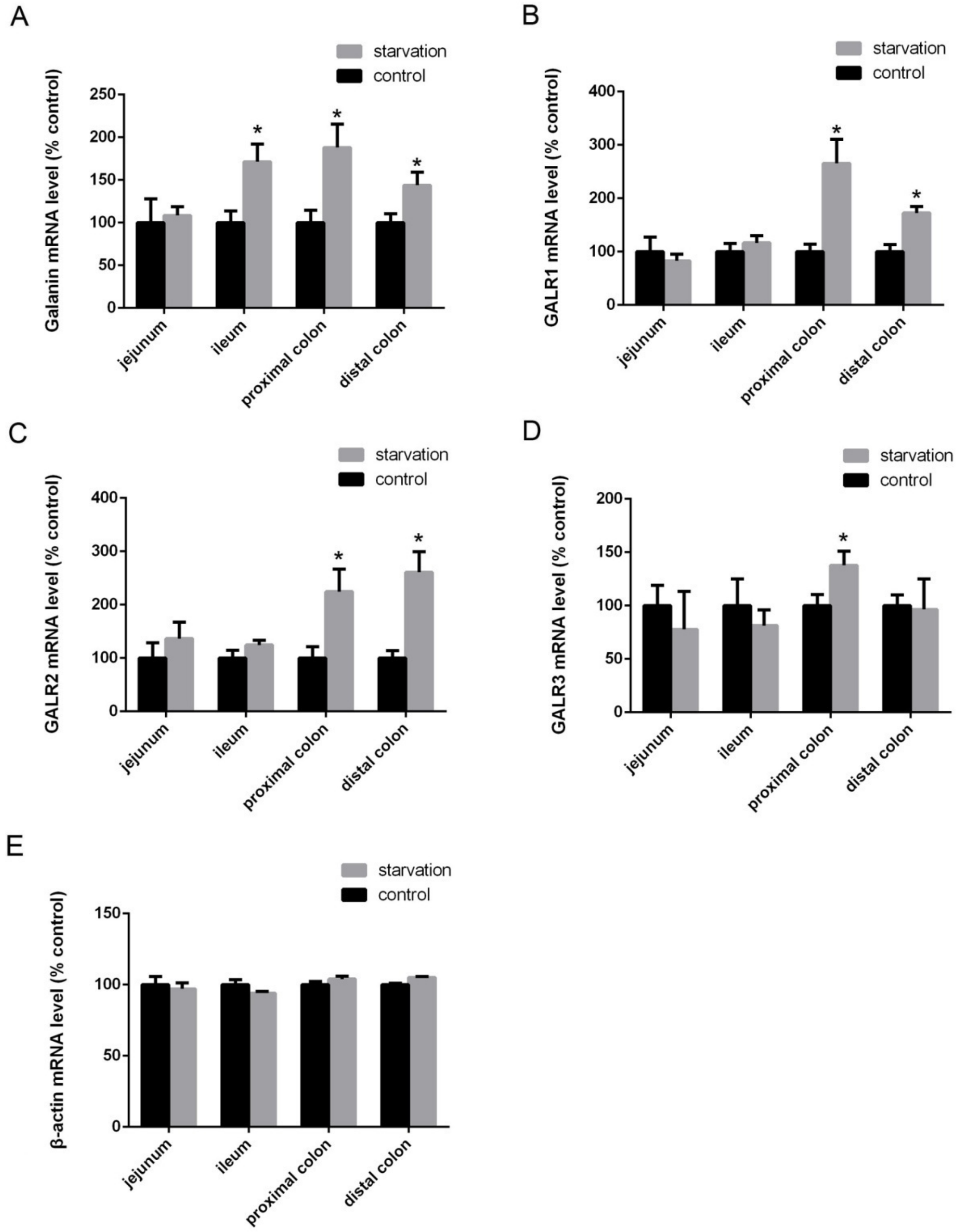

Supplementary Figure S6. The mRNA levels of galanin and galanin receptors in intestine and colon of mice under the starvation condition. Mice were starved for 24 hours and then the total RNA of proximal colon, distal colon, jejunum and ileum were isolated, cDNA was synthesized and quantitative real-time PCR for galanin (A), GALR1 (B), GALR2 (C) and GALR3 (D) were conducted. The β-actin (E) showed no significant difference between groups as an internal control. Each group contained 8 mice. Statistical differences between individual groups were evaluated using One way ANOVA. *, P＜0.05 compared with paired saline-treated controls.

Supplementary Table S1. The energy of best docking pose in mGALR2/3-Spexin complexes

Complex	*Binding free energy (GBVI/WSA dG, kcal/mol)*	*Ligand conformational energy (kcal/mol)*
mGALR2-Spexin	-21.30	-312.11
mGALR3-Spexin	-17.22	-344.32

Supplementary Table S2. Important Residues Involved in the mSPX-mGALR2/3 Interactions

mSPX	*mGALR2*		*mGALR3*	
	Residue	*Interaction type*	*Residue*	*Interaction type*
Asn1	na	na	Glu177	HB
Trp2	Phe193, Ile274	HC*	Phe78,Trp88	HC
Gln5	Gln81	HB**	na	na
Met7	Trp20	HC	Ile82,Leu85,Trp88	HC
Leu8	Trp20	HC	na	na
Lys11	na	na	Leu169, Leu171	HB
Gly12	Gln167	HB	na	na
Gln14	S166, S179, R184	HB	Ser266	HB

*HC,hydrophobic contact; **HB, hydrogen bonding; na, not available.

Supplementary Table S3. Primer sequences for qRT-PCR for mouse galanin and galanin receptors

Genes	Abbreviations	Sequence
Galanin	Galanin	5'-GTGACCCTGTCAAGCCACTCT-3'<br>5'-GGTCTCCTTTCCTCCACCTC-3'
Galanin receptor 1	GALR1	5'-GCCGCGATGTCTGTGGATCG-3'<br>5'-CGATGGACAGCGCCCAGATG-3'
Galanin receptor 2	GALR2	5'-GTGTGCCACCCAGCGTGGAG-3'<br>5'-TGGTGCGCGCATAGGTCAGG-3'
Galanin receptor 3	GALR3	5'-TTCGTGTGCAAGACGGTACA-3'<br>5'-TTAGGTAGGGCGCGGAAAAG-3'

Supplementary Table S4. Inter-assay variation determined by running standard curves on 7 different assay kits (supplied by Phoenix Pharmaceuticals, Inc.).

Sample	Concentration	Values	Mean Value	Std. Dev.	CV%
St01	0.01	2.578, 2.302, 2.4, 2.402, 2.598, 2.469, 2.292	2.434	0.082	5
St02	0.1	2.024,1.924,1.846, 1.979, 1.912, 1.936,1.778	1.914	0.082	4.3
St03	1	0.647,0.657,0.614,0.628, 0.621,0.642,0.534	0.62	0.041	6.6
St04	10	0.647,0.657,0.614,0.628, 0.621,0.642,0.534	0.146	0.019	13.3
St05	100	0.065,0.113,0.093,0.108, 0.087,0.075,0.093	0.091	0.017	18.7

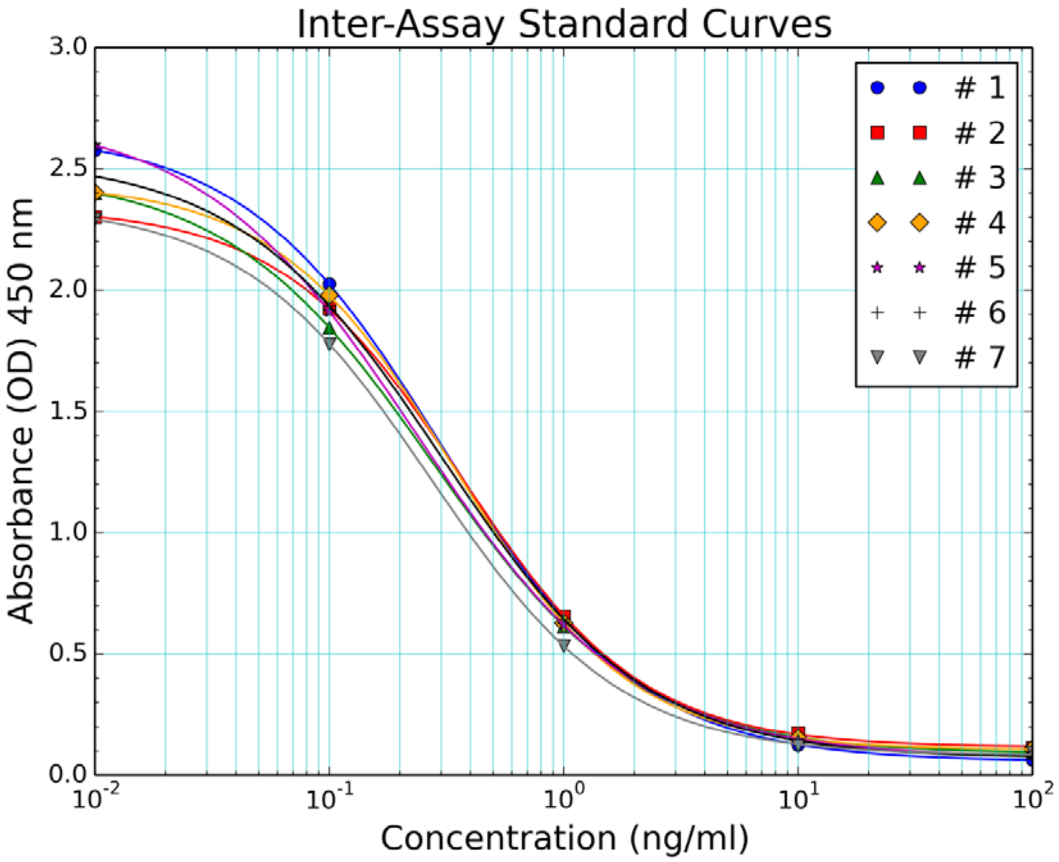

Log-Logit Fit: y=(A-D)/(1+(x/C)^B)+D:	A	B	C	D	R^2
Standard curve #1	2.58	1.09	0.33	0.065	0.998
Standard Curve #2	2.3	1.14	0.385	0.113	0.997
Standard Curve #3	2.4	1.04	0.308	0.093	0.996
Standard Curve #4	2.4	1.18	0.355	0.108	0.999
Standard Curve #5	2.6	1.03	0.274	0.087	0.995
Standard Curve #6	2.47	1.05	0.332	0.075	0.997
Standard Curve #7	2.29	1.15	0.294	0.093	0.996

Supplementary Table S5. Intra-assay variation determined by running standard curves on 7 different assay kits (supplied by Phoenix Pharmaceuticals, Inc.).

Sample	Concentration	Values	Mean Value	Std. Dev.	CV%
St01	0.01	2.945, 2.585, 2.66, 2.666, 2.388, 2.388, 2.299	2.562	0.223	8.7
St02	0.1	2.24, 2.466, 2.169, 2.509, 2.513, 2.166, 2.066	2.304	0.187	8.1
St03	1	0.866, 0.852, 0.663, 0.801, 0.813, 0.822, 0.791	0.801	0.067	8.3
St04	10	0.206, 0.256, 0.251, 0.21, 0.21, 0.227, 0.196	0.222	0.023	10.5
St05	100	0.158, 0.188, 0.189, 0.185, 0.156, 0.156, 0.201	0.176	0.019	10.7

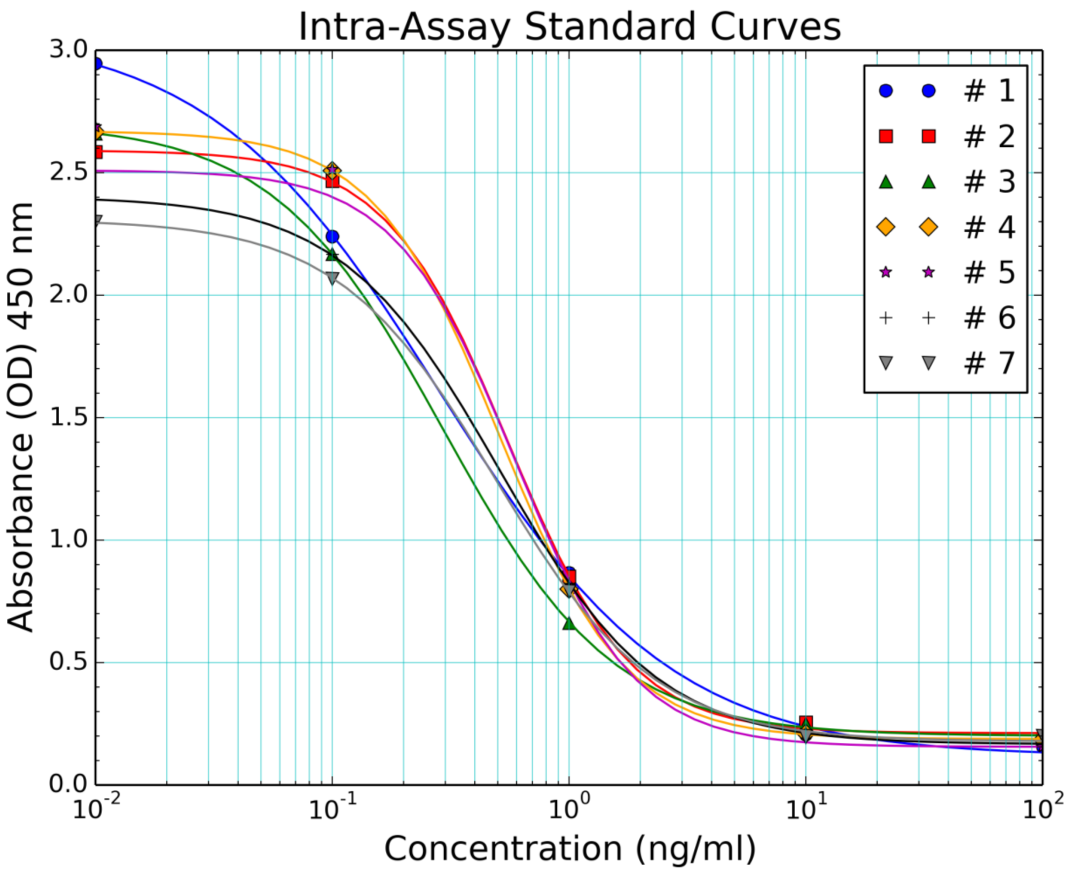

Log-Logit Fit: y=(A-D)/(1+(x/C)^B)+D:	A	B	C	D	R^2
Standard curve #1	2.94	1.03	0.327	0.158	0.985
Standard Curve #2	2.58	1.21	0.524	0.188	0.978
Standard Curve #3	2.66	1.2	0.31	0.189	0.993
Standard Curve #4	2.67	1.63	0.507	0.185	0.999
Standard Curve #5	2.51	1.71	0.588	0.156	0.911
Standard Curve #6	2.39	1.24	0.515	0.156	0.993
Standard Curve #7	2.3	1.43	0.51	0.196	0.973

Supplementary Methods

Building the Mouse Spexin Model

Homology Modeling

The mouse spexin (mSPX) is a 14AA peptidewhich share the identical sequence (NWTPQAMLYLKGAQ) with human spexin (hSPX) and goldfish spexin (gSPX). The solution structure of gSPX was studied by nuclear magnetic resonance (NMR), which showed that there was an α-helix spanning from Gln5 to Gln14[1]. Although there was no NMR models deposited into the public database, we built the initial structure of mSPX by according to the structural features of gSPX model with Protein Builder module in MOE[2].The 3D model of mSPX was amidated at the C-terminus. It was further minimized with Amber12:EHT forcefield and R-Field solvation model. The secondary structures (SS) of the first four residues in mSPX initial model are random coil, and the remaining are majorly α-helix.

MD Simulation

The initial structure of mSPX was further relaxed in all-atoms, explicit water,10 ns MD simulation with ff12SB forcefield. Initially, it was solvated into a periodic boundary, cubic, and TIP3P[3] explicit water box with a 12 Å buffer distance by LEaP module in Amber Tools 14[4].The charge of whole system was neutralized by adding counter ions.

The prepared system was minimized and equilibrated by sanderin three stages: (1) heating from 100 K to 300 K in 20 ps; (2) adjusting solvent density to 1 g/mL in 20 ps; (3) further equilibrating in 200 ps with constant pressure and constant temperature (NPT). Then, 10 ns, and NPT production simulation was carried out by CUDA-accelerated PMEMD[5]. A 2 fs time step was used and bonds involving hydrogen were constrained by SHAKE algorithm[6] for all equilibration and production stages.

Trajectory Analysis

The trajectories during the production stage were analyzed by cpptraj[7]. To calculate the RMSD of protein or spexin, the C_α was used for structural superposition. The RMSD value was calculated by comparing with the initial and end structures for all snapshots during the production stage. Snapshots captured from 10 ps interval were clustered on C_α RMSD to generate clusters using average-linkage, stopping when either 3 clusters are reached or minimum distance between clusters is 4.0. The representative conformation from the biggest cluster was used for next step.

Building the Mouse GLAR1/2/3 Model

Template Choosing

The mouse GALR2/3 protein sequences (UniProtID,O88854 and O88853) were retrieved from UniProt (http://www.uniprot.org). Those sequences were submitted to PDB Bank (http://www.rcsb.org/pdb) to search for the appropriate template by using BLAST (low complexity mask='Yes', e-value cutoff=10.0, sequence identity cutoff=0). Among the searched results, 4EA3, the structure of the human N/OFQ receptor (OPRL1) in complex with a peptide mimetic[8], was of the most interest because: (1) OPRL1 and GALR1/2/3 belong to the same GPCR subfamily A; (2) the resolved OPRL1 structure owned reasonable aligned sequence length (254 and 278) and the best sequence identity (31% and 28%) with GALR2/3. Taken all above, the crystal structure of OPRL1 was chosen as template in building homology models for mouse GALR2/3.

Sequence Alignment

The pdb file was downloaded and imported into MOE, only chain A and the bound ligand were kept and others were removed. The mouse GALR2/3 sequences were also imported and aligned with 4EA3 chain A in presence of GPCR constrains.

Homology Modeling

The models of mouse GALR2/3 were built by following standard homology modeling proceduresin MOE. The C-terminal and N-terminal modeling were disabled. Fifty intermediated models were generated in the process of mainchain and sidechain sampling. Medium refinement was chosen for both intermediates and final model refinement with the Amber12:EHT forcefield and R-field solvation model. The hydrogens were added by Protonate3D[9] before refining the final model. The final model was chosen based on the Generalized Born/Volume Integral (GB/VI) electrostatic solvation energy[10].

MD Simulation

To further refine the model and remove unreasonable contacts, we carried out 10 ns MD simulation for mouse GALR2/3 in the cell membrane environment, built by Membrane Builder in CHARMM-GUI[11]. Firstly, the homology model of mouse GALR2/3 in PDB format were uploaded to the CHARMM-GUI website. Secondly, the whole protein was aligned by the first principal axis along Z. The heterogeneous lipids, POPC and POPE, were added into upper leaflet and lower leaflet. The 0.15 M KCl was also included to neutralizing the charge of whole system with Monte-Carlo placing method. Finally, the prepared membrane system was transformed for MD simulation in Amber 14. The prepared system was generated by LEaP in AMBER 14. The ff12SB and Lipid14[12] were chosen as forcefield for protein and lipids, respectively.

To equilibrate and simulate the mouse GALR2/3 membrane protein systems, there were 4 stages: (1) heating to 100 K in 5 ps; (3) heating system from 100 K to 303 K in 100 ps; (4) equilibrating system by repeating 10 times hold protocol, 500 ps for each time; (5) 10 ns, NPT production simulation. The 2fs time step and SHAKE algothrim[6] were used in all equilibration and production stages.

Trajectory Analysis

The trajectory analysis method was same as the one used in building mSPX model. The representative conformations of mouse GALR2/3 from the biggest cluster was used for next step.

Building the Mouse GLAR/2/3-Spexin Complex Models

Defining the Binding Site

At first, the small molecule (1-benzyl-N-[3-(1'H,3H-spiro[2-benzofuran-1,4'-piperidin]-1'-yl)propyl]-D-prolinamide), which was complexed with OPRL1 in the template, was used as reference ligand to define the binding site for molecular docking. However, compared with mSPX, it was too small to represent a proper binding site for peptides. After searching the PDB database, we found a structure that the CXCR4 chemokine GPCR complexed with 16 AA long peptide CVX15 (PDB ID, 3OE0)[13], where the ligand was of a close size with mSPX. By superposing this receptor with mouse GALR2/3 models, CVX15 was used as reference ligand to define the possible binding site of mSPX.

Flexible Docking

The flexible docking were carried out in MOE with Dock module. The mSPX was defined as ligand and the mGALR2/3 was defined as receptor. The mGPX was kept rigid, proxy triangle was used as placement method and London dG was used for scoring at the first stage. The docking poses were further refined with molecular mechanics for both mSPX and receptor residue 8 Å around the mSPX. The final poses were scored by GBVI/WSA dG scoring function. In both the placement and refinement stages, at most 30 poses were retained.

Analyzing the Docking Results

The docking results were analyzed based on the binding free energy, ligand conformational energy, as well as the important contacts from prior knowledge. The best pose was used for analyze the important interactions between mSPX and mGALR2/3.

Supplementary Results

The Mouse Spexin Model

In a 10 ns MD simulation, the overall conformation of mSPX in the water solution was stably evolved. Compared with the initial structure, the C_α RMSD of mSPX residues, except the flexible N- and C-terminus residues, were between 1 and 3 Å (Fig. S2A).The representative conformation of mSPX showed distinct secondary structure features: (1) the N-terminal residues, Asn1, Trp2, and Thr3 were coiled randomly; (2) the residues at the middle, Pro4, Gln5, Ala6, Met7, Leu8, Tyr9, and Leu10 were in alpha helix; (3) the remaining residues at the C-terminal, Lys11, Gly12, Ala13 and Gln14 were in random coil (Fig. S2B). It was worth noting that, although in the same secondary structure, the N-terminal residues are more rigid than the C-terminal residues, with the backbone C_α root mean structural fluctuation (RMSF) less than 1.5 Å as compared to 2 to 4 Å (Fig. S2C).

The Mouse GALR2/3 Model

The mGALR2/3 is belong to the G protein-coupled receptors (GPCR) superfamily and class A subfamily. To align the sequence of mGALR2/3 with the template 4EA3 chain A, the seven transmembrane (TM) domains of both query and template sequences were annotated firstly by comparing with a database of GPCR in MOE. The whole sequences were aligned with emphasizing the importance of TM residues (Fig. S3). From the alignment results, the disulfide bonds were predicted: C98 and C174 in mGALR2, C95 and C172 in mGALR3. Those disulfide bonds were kept in homology modeling to ensure the intact structures. Due to lack of structural information in template, the N-terminal outgaps (M1-G19 in mGALR2, M1-P12 in mGALR3) and the C-terminal outgaps (R315-C371 in mGALR2, L305-Q370) were omitted in homology modeling.

The conformation of mGALR2/3 within membrane environment was stable in 10 ns MD simulation, with C_α RMSD around 2 Å (Fig. S4A). The overall structure of representative conformation of mGALR2/3 show distinct GPCR structural features: the 7 TM helixes crossed the cell membrane and were linked with several loops in the exterior cell (EC) and inner cell (IC) (Fig. S4B). The binding cavity was also clear shown. The EC loop linking TM4 and TM5, the IC loop linking TM5 and TM6, and the EC loop linking TM6 and TM7, were of the most flexibility (Fig. S4C).Those results suggested that the binding cavity for ligands could be flexible and the induce-fit binding may happen.

The Mouse GALR2/3-Spexin Complexes Model

By using flexible docking, the mGALR2/3-Spexin complex models were generated. Firstly, we filtered the docking poses with prior knowledge. Galanin was the first identified endogenous ligand for GALRs and the binding between galanin and GALRs were extensively studied. It was found that the N-terminal residues (1-16) were essential for binding and activate the GALRs in rat[14]. The Trp2of galanin was found extremely important for binding all GALRs in rat[15].Since it is also conserved in spexin, we postulated that the N-terminal of mSPX, especially Trp2 is important for binding with mGALR. The docking poses were observed manually, and if Trp2 was not inserted into the binding cavity, such poses were excluded. The remaining poses were ranked by binding free energy (index for binding strength) and the ligand conformation energy (index for the strain energy of ligand) and the best poses were used for interaction analysis.

From the overall view of mGALR2/3-Spexin best docking poses, the mSPX fitted the binding site of mGALR2/3 well (Fig. 5SA). In the best docking pose of mGALR2-Spexin, the calculated binding free energy was -21.30 kcal/mol, while it was -17.22 kcal/mol in mGALR3-Spexin (Table S1). The relative magnitude was in line with the EC50 of spexin toward human GALR2 (161 nM) and GALR3 (626 nM)[16].However, the conformational energy of mSPX in mGALR2-Spexin best pose was -312.11 kcal/mol, while it was -344.20 kcal/mol in mGALR3-Spexin best pose (Table S1). Such difference reflected that the unbound conformation of mSPX was slightly changed in mGALR3-Spexin complex, but largely changed in mGALR2-Spexin complex (Fig. S5A).

Several residues involved in the mGALR2/3-Spexin interactions could be predicted from the best docking poses (Table S2). The hydrophobic contacts formed with Trp2 and Met7 in mSPX were conserved in both mGALR2- and mGALR3-Spexin complexes. Those results implied the important role of hydrophobic residues of mSPX in receptor binding. Hydrogen bonding may also form between other residues of mSPX and residues in binding cavity or EC loops. Although they need to be confirmed by binding assay and mutation experimentation, these results suggested that mSPX may also bind and activate mGALR2/3.

Supplementary Reference

1. Wong, M. K. H. *et al.* Goldfish spexin: solution structure and novel function as a satiety factor in feeding control. *Am. J. Physiol. Endocrinol. Metab.***305,** E348–66 (2013).

2. Molecular Operating Environment (MOE) v. 2012.10 (Chemical Computing Group Inc. 1010 Sherbooke St. West, Suite #910, Montreal, QC, Canada, H3A 2R7, 2012).

3. Jorgensen, W. L., Chandrasekhar, J., Madura, J. D., Impey, R. W. & Klein, M. L. Comparison of simple potential functions for simulating liquid water. *J. Chem. Phys.***79,** 926 (1983).

4. D.A. Case, V. Babin, J.T. Berryman, R.M. Betz, Q. Cai, D.S. Cerutti, T.E. Cheatham, III, T.A. Darden, R.E. Duke, H. Gohlke, A.W. Goetz, S. Gusarov, N. Homeyer, P. Janowski, J. Kaus, I. Kolossváry, A. Kovalenko, T.S. Lee, S. LeGrand, T. Luchko, R. Luo, B., X. W. and P. A. K. AMBER. (2014).

5. Götz, A. W. *et al.* Routine microsecond molecular dynamics simulations with AMBER on GPUs. 1. generalized born. *J. Chem. Theory Comput.***8,** 1542–1555 (2012).

6. Ryckaert, J.-P., Ciccotti, G. & Berendsen, H. J. . Numerical integration of the cartesian equations of motion of a system with constraints: molecular dynamics of n-alkanes. *J. Comput. Phys.***23,** 327–341 (1977).

7. Roe, D. R. & Cheatham III, T. E. PTRAJ and CPPTRAJ: software for processing and analysis of molecular synamics trajectory data. *J Chem Theory Com***9,** 3084–3095 (2013).

8. Thompson, A. a. *et al.* Structure of the nociceptin/orphanin FQ receptor in complex with a peptide mimetic. *Nature***485,** 395–399 (2012).

9. Labute, P. Protonate3D: assignment of ionization states and hydrogen coordinates to macromolecular structures. *Proteins***75,** 187–205 (2009).

10. Labute, P. The generalized Born/volume integral implicit solvent model: estimation of the free energy of hydration using London dispersion instead of atomic surface area. *J. Comput. Chem.***29,** 1693–8 (2008).

11. Jo, S., Kim, T., Iyer, V. G. & Im, W. CHARMM-GUI: a web-based graphical user interface for CHARMM. *J. Comput. Chem.***29,** 1859–65 (2008).

12. Dickson, C. J. *et al.* Lipid14: The amber lipid force field. *J. Chem. Theory Comput.***10,** 865–879 (2014).

13. Wu, B. *et al.* Structures of the CXCR4 chemokine receptor receptor in complex with small molecule and cyclic peptide antagonists. *Science (80-.).***330,** 1066–1071 (2010).

14. Fisone, G. *et al.* N-terminal galanin-(1-16) fragment is an agonist at the hippocampal galanin receptor. *Proc. Natl. Acad. Sci. U. S. A.***86,** 9588–9591 (1989).

15. Wang, S., He, C., Hashemi, T. & Bayne, M. Cloning and Expressional Characterization of a Novel. 31949–31952 (1997).

16. Kim, D. K. *et al.* Coevolution of the spexin/galanin/kisspeptin family: Spexin activates galanin receptor type II and III. *Endocrinology***155,** 1864–1873 (2014).
